# Clinical Impact of Thermotherapy and Spinal Twisting Massage on Chronic Non-Specific Spinal Pain

**DOI:** 10.3390/medicina60060976

**Published:** 2024-06-13

**Authors:** Syung Hyun Cho, Un Mo Jeong, Sung Hoon Kim

**Affiliations:** 1 Department of Medicine, Wonju College of Medicine, Yonsei University, Wonju 26426, Gangwon-do, Republic of Korea; nugabest@nuga.kr; 2 Department of Biomedical Engineering, Yonsei University, Wonju 26493, Gangwon-do, Republic of Korea; jungwoonmo@gmail.com

**Keywords:** chronic non-specific spinal pain, spinal twist massage, heat, Korean Western Ontario and McMasters Universities, Visual Analog Scale

## Abstract

As the prevalence of chronic non-specific spinal pain rises, the utilization of diverse massage devices for therapeutic intervention increases rapidly. However, research on their mechanisms, particularly those involving spinal twisting, is limited. This study was designed to evaluate the impact of heat application and spinal twisting massage techniques on individuals suffering from chronic non-specific spinal pain. A total of 36 individuals were divided into two groups: a control group (18 participants) and an experimental group (18 participants). The experimental group received heat treatment plus spinal twisting massage twice a week for four weeks, while the control group received heat therapy plus traditional vibration massage techniques. Effectiveness was measured using the Visual Analog Scale (VAS), the Pressure Pain Threshold (PPT), the Korean Western Ontario and McMaster Universities (K-WOMAC) Index, spine tilt, and Cobb angle. VAS, K-WOMAC, and PPT significantly improved in both groups at all three time points. VAS notably decreased in the experimental group compared to the control group (*p*-value: 0.0369). Despite improvements in K-WOMAC and PPT scores within the experimental group, statistical significance remained elusive. Furthermore, spine tilt and Cobb angle showed no significant differences from baseline to the 6th week. In conclusion, the application of thermotherapy coupled with twisting massage demonstrates significant efficacy in mitigating chronic non-specific spinal pain, surpassing the pain-relief outcomes achieved through heat therapy in combination with standard vibration massage techniques.

## 1. Introduction

In contemporary society, a considerable number of individuals predominantly adopt sedentary lifestyles, leading to spinal issues and discomfort due to improper posture and heightened stress levels. This trend is evident in the increasing number of patients seeking medical attention, resulting in a growing demand for healthcare services [1]. According to Cieza’s 2019 study on the global burden of diseases, injuries, and risk factors, chronic spinal pain (including low back pain) remains the most prevalent health issue worldwide, significantly contributing to disability [2]. Alongside a decrease in physical activity, these issues related to spinal pain exert a substantial impact on daily life, causing economic burdens such as reduced productivity, increased absenteeism, and heightened healthcare expenditures, extending beyond individual physical concerns [3,4].

There are spinal diseases with clear causes, such as spinal stenosis, posterior joint syndrome, and spinal disc dislocation [5]. However, a significant number of patients experience non-specific spinal pain, which presents diagnostic challenges due to its multifaceted etiology [6]. The etiology of non-specific spinal pain, which disproportionately affects women compared to men, remains unclear. Consequently, a wide range of therapeutic approaches are employed, with the primary goal of symptom reduction [6].

This study aims to assess the clinical efficacy of STMDs, integrating both heat therapy and spinal twisting massage, among individuals afflicted with chronic non-specific spinal pain. Our primary objective is to validate the pain-alleviating properties of administering spinal thermal massage to individuals afflicted with chronic non-specific spinal pain. Our secondary goal is to compare the effectiveness of heat therapy in conjunction with vibrating massages with spinal thermal massage to assess its therapeutic efficacy.

Conservative approaches are commonly employed for alleviating spinal pain, and these can be broadly classified into physical and non-physical therapies. Physical therapies involve employing modalities such as heat, cold, massage, ultrasound, low-level laser, and electrical stimulation. In contrast, non-physical therapies for spinal pain include pharmacological treatment, cognitive–behavioral therapy, and more. In addition to the mentioned treatment methods, commonly employed pathological treatment options include electrical therapy, injection therapy, and more [7,8].

Heat therapy is considered to enhance metabolism and promote circulation by dilating blood vessels and improving enzyme functions. These actions result in increased catabolism, the excretion of lactic acid, free fatty acids, and subcutaneous fat, as well as the removal of uric acid and other acidic waste products from muscle cells. Through these mechanisms, heat therapy is believed to reduce fatigue and signs of aging, producing an analgesic effect. Clinically, heat treatment has been proven effective in managing various injuries and providing pain relief for muscles and skeletal structures such as the back, shoulders, and waist. This has led to its diverse treatment applications, demonstrating its efficacy [9]. Massage therapy is a widely recognized therapeutic approach that can be utilized to address a variety of musculoskeletal pains including non-specific spinal pain [10]. Its historical usage extends across diverse civilizations, including significant practice in Asia, particularly in China and India, and traces back to ancient Egyptian civilizations [11]. In a comprehensive database review conducted by Furlan and colleagues in 2015, massage therapy exhibited substantial advantages in providing short-term pain relief and functional improvement for individuals with both acute and chronic spinal pain when compared to inactive control interventions [12].

Recently, a Spinal Thermal Massage Device (STMD), designed to provide simultaneous heat and massage therapy, has been introduced to the market and is gaining widespread use. This innovative medical device addresses a significant gap in non-invasive therapeutic options for relieving back pain, a condition affecting millions globally. By combining heat therapy with precise mechanical massage, the STMD offers potential relief by enhancing blood circulation, reducing muscle tension, and promoting spinal health [13,14,15]. Understanding its efficacy through rigorous scientific studies is essential to providing invaluable insights into its benefits and limitations and ensuring its safe integration into clinical practice. Previous studies have shown that the device provides pain relief in the shoulder, pelvic, and neck regions [16]. Additionally, research conducted by Lee et al. found that one month of STMD usage positively impacted depression and stress reduction [17]. Several other studies have also examined non-invasive and alternative treatments [18,19,20,21], highlighting the need for comprehensive clinical trials to establish STMDs’ wide-ranging efficacy on pain relief, functional mobility, and overall spinal health across diverse patient populations. This research bridges the gap between innovative technology and practical, accessible healthcare solutions, promising a future where spinal pain can be managed more effectively, safely, and economically.

## 2. Materials and Methods

### 2.1. Participants

This study was conducted from 21 June 2023 to 23 September 2023 in accordance with the Declaration of Helsinki and approved by the Institutional Review Board of Yonsei University Wonju Christian Hospital (IRB No. CR223008). Informed consent was obtained from all participants involved in this study. All participants were provided with comprehensive information about the purpose and methods of this study and voluntarily signed consent forms before its commencement.

Participants were recruited from patients diagnosed with chronic non-specific spinal pain, aged 20 to 80 years. Through screening, individuals with a Visual Analog Scale (VAS) score of at least 40 mm (max. 100 mm) and a Korean Western Ontario and McMasters Universities (K-WOMAC) score of 30 or higher were included. Those with a history of spinal procedures or surgery within the last 3 months, individuals with chronic inflammatory conditions such as ankylosing spondylitis, and those with the potential for interference with treatment efficacy due to other diseases were excluded from this study. However, patients with limb dissymmetry were not excluded from this research.

The sample size for the clinical trial assessing clinical efficacy was calculated [22] to be 16 participants per group, with a consideration of a 10% dropout rate, resulting in a final sample size of 18 participants per group. Consequently, a total of 36 patients (7 males and 29 females, mean age: 64.97 ± 8.7) diagnosed with chronic non-specific spinal pain participated in our study, and there were no dropouts during our research. The characteristics of the participants are shown in Table 1.

### 2.2. Device

**Figure 1:**
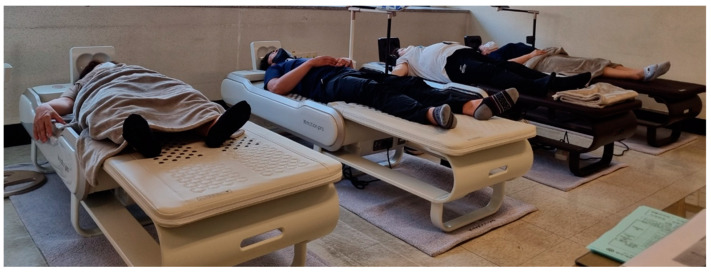
Treatment device and how to use it (NUGA MEDICAL, N7^®^).

To evaluate the effectiveness of the mechanical heat and spinal twisting massage, this study utilized the combinational stimulator N7 (NUGA Medical Co., Wonju-si, South Korea) as the treatment equipment, as shown in Figure 1. For the control group, the same model was used, but with the twisting massage protocol removed; only vibration massage through simple horizontal/vertical movements of the massage roller was provided. The spinal twisting massage function involved a combination of spinal traction through the up-and-down movement of the massage rollers and a twisting pressure applied to the spinal joints in a thumb-like massage pattern, facilitating spinal rotation. The traction height difference due to the up-and-down movement was 45 mm, and the twisting motion provided an additional rotational pressure of approximately 12 mm. The equipment was operated by the examiner and automatically operated for 30 min in the selected mode to provide heat and massage to the subjects.

### 2.3. Procedures

Participants who met all the inclusion and exclusion criteria were assigned by the investigator in the order of registration and randomly allocated to either the experimental or control group in a 1:1 ratio, as shown in Table 2. Randomization envelopes were then prepared with the issued randomization numbers and provided to the unblinded investigator at the institution until participant registration. In this trial, the roles of the investigator were divided into the non-blinding practitioner, responsible for random participant allocation and application, and the blinded assessor, responsible for evaluating the outcomes. Participants received blinding regarding group allocation and the application method of the massage, with no information provided. At the initial visit, the 36 selected participants were assigned registration and random allocation numbers. They were then divided into two groups: the experimental group and the control group. Different treatments were applied to each group. The experimental group received heat and twisting massage, while the control group received heat and simple vibration massage. The study design was informed by a thorough review and integration of pertinent findings from the existing research literature, establishing a robust foundation for this research [18,20,21,22,23,24,25]. The characteristics of the cont. vs. exp. groups are shown in Table 3.

**Figure 2:**
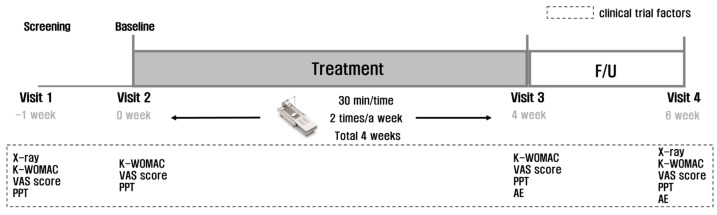
Treatment and test protocol.

According to the test protocol (Figure 2), the participants underwent a total of 8 heat and massage sessions over 4 weeks (30 min per session, twice a week). Evaluations of safety and efficacy were conducted at 4 and 6 weeks after the initial application, and clinical results were collected and analyzed at the end of the 6th week’s visit for all participants.

During the clinical trial period, the use of oral or non-oral corticosteroids; NSAIDs (nonsteroidal anti-inflammatory drugs); and concurrent surgery, medication, or other physical therapy or medical devices that could affect safety, efficacy evaluation, or clinical trial results were controlled and prohibited.

In cases where participants experienced unbearable pain during the clinical trial period, rescue medication was allowed. The rescue medication could be taken every 6 h, and since acetaminophen could cause liver damage if the maximum daily dose of 2600 mg was exceeded, participants were educated not to exceed 4 tablets within 24 h, and their medication records were checked. Considering the potential impact and bias of acetaminophen on efficacy evaluation, a washout period of at least 5 times the biological half-life (1~4 h) was set, that is, a 24 h washout period (half-life 4 h X 6). Participants were instructed not to take rescue medication 48 h before Visits 2, 3, and 4, where efficacy evaluations were measured. Additionally, participants were required to self-assess VAS before taking rescue medication and record it in their diaries.

For the clinical efficacy assessment of this study, variables such as the VAS for pain, Pressure Pain Threshold (PPT) for pain, and the K-WOMAC were used to evaluate the daily living functions of the subjects. Additionally, general radiography of the spine was performed at the time of research screening and at the last visit of our study to serve as a secondary assessment measure for the structural aspects of the spine.

#### 2.3.1. Monitoring the Changes in VAS

To assess the changes in spinal pain at 4 and 6 weeks compared to the baseline, a 100 mm VAS was used. This scale measures pain intensity, with 0 indicating no pain and 100 representing intolerable pain. Participants pointed to a position on the scale that corresponded to their perceived level of pain [26].

#### 2.3.2. Monitoring the Changes in PPT

PPT using a pressure algometer is effective in exploring physio-pathological mechanisms and is reliably utilized in clinical settings [27,28,29]. The measured values of PPT serve as an indicator of the subjective intensity of pain perceived by the individual, making it effective for monitoring individual variations in pain levels over time. Several studies generally indicate that females tend to exhibit lower thresholds compared to males [30,31,32].

**Figure 3:**
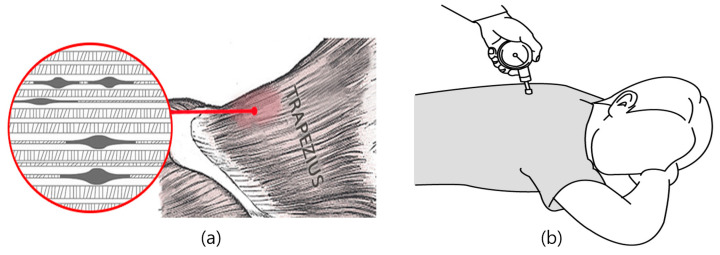
(**a**) Examples of pain-triggering points in the muscle; (**b**) measuring PPT using a pressure algometer.

In this study, at baseline (week 0) and subsequently at 4 and 6 weeks, the alteration in pressure at the most sensitive area of the spinal pain trigger points was gauged three times using a pressure gauge at the same location for each time point are shown in Figure 3. This method quantifies the pressure change at the site of the greatest pain, providing an objective measure of pain intensity and its variation over time.

#### 2.3.3. Monitoring the Changes in K-WOMAC Scores

The WOMAC is a tool for clinically evaluating changes in functional status related to joints and is applicable for assessing clinical treatment outcomes in patients with non-specific spinal pain. By providing questions related to spinal disorders, it enables a detailed assessment of the degree of specific functional limitations and integrates the evaluation of the extent of limitations in specific functions related to pain and disability. Therefore, it is recognized as a specific indicator for assessing spinal pain and functional improvement [33].

**Figure 4:**
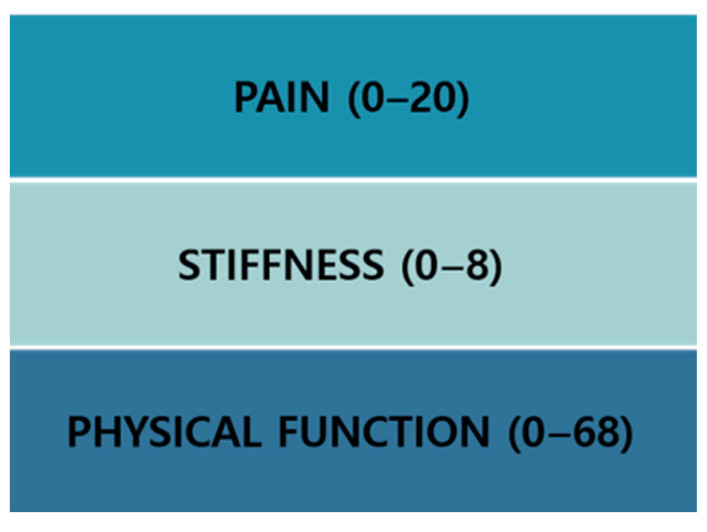
**K**-WOMAC scores.

The K-WOMAC Index, translated by Bae et al. and validated for its reliability, validity, and responsiveness, serves as a survey tool for pain-related diseases [34]. It is primarily applied in clinical practice to assess functional status. The K-WOMAC Index is widely adopted due to its instrumental validity, sensitivity to change, and reported high reliability as shown in Figure 4.

**Figure 5:**
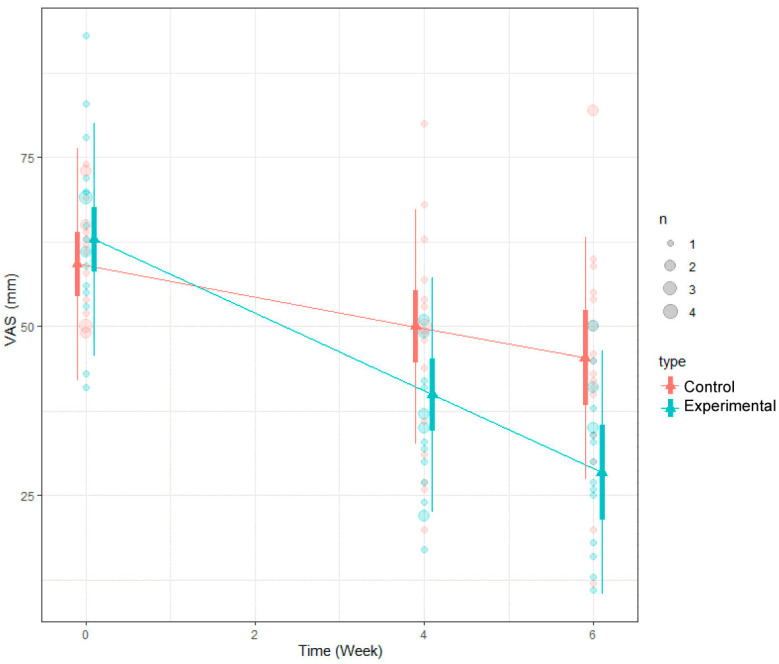
LMM analysis results of VAS.

**Figure 6:**
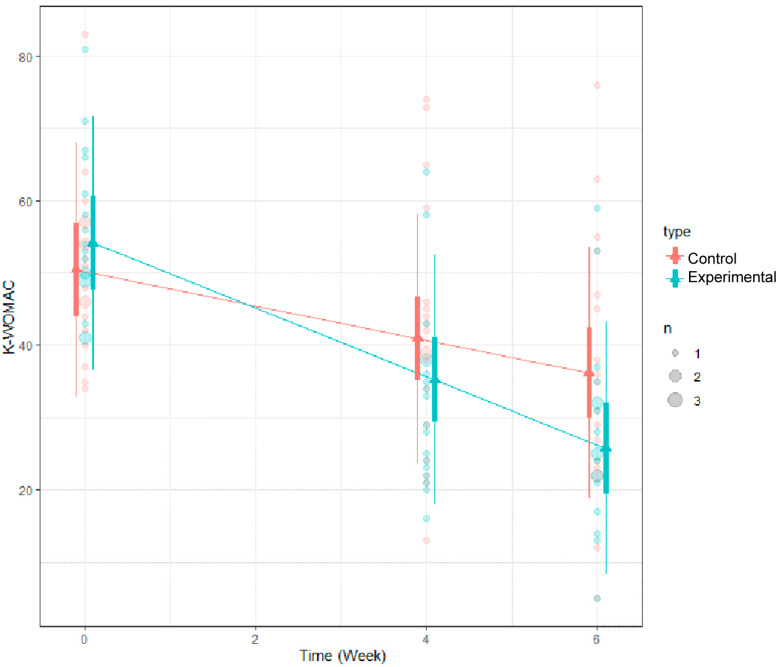
LMM analysis results of K-WOMAC.

The K-WOMAC evaluation comprises questions related to functional limitations associated with pain. It consists of 24 questions and three subscales, and it includes 5 questions about pain, 2 questions about stiffness, and 17 questions about physical function. This assessment is fundamentally used in the field of rehabilitation medicine to evaluate pain and function. Moreover, it is applied in assessing basic daily life abilities and pre- and post-evaluations of pain. In this study, it was utilized to assess participants’ daily life abilities and pain, among other aspects, through pre- and post-evaluations (Figure 5 and Figure 6).

In this study, the K-WOMAC was used to evaluate changes in pain, physical function, and stiffness index at 4 and 6 weeks compared to baseline (0 weeks).

#### 2.3.4. Monitoring the Changes in Spinal Tilt Angle and Cobb Angle

The measurement of spinal inclination angle and Cobb angle is crucial in evaluating the structure and posture of the spine, serving as important clinical indicators. Measurements using X-ray imaging assess the curvature and alignment of the spine, and this method is reported to have high reliability for such evaluations [35].

At the 6-week mark compared to the baseline (week 0), changes in the sacral inclination angle and the scoliosis angle, measured using the Cobb method, were assessed through general radiographic imaging of the spine. The objective of this assessment was to ascertain anatomical variations in the spinal region, specifically in alignment and curvature, following the implementation of treatment. The Cobb method, a conventional technique for quantifying spinal deformities, especially in the context of scoliosis, was employed in the radiographic analysis.

### 2.4. Statistics

For the statistical analysis of this study, we applied the Statistical Analysis System (version 9.4, SAS Institute Inc., Cary, NC, USA) and R Studio (version 4.1.3, R Studio, Inc., Boston, MA, USA). Continuous data from all participants in this clinical trial were presented with mean, standard deviation, median, minimum, and maximum values. For categorical data, we provided frequencies and proportions. Between the experimental group and the control group, group-wise differences were assessed using the two-sample t-test (or Wilcoxon’s rank sum test if the data did not meet the normal distribution assumption) for continuous variables. For categorical variables, the comparison was performed using Pearson’s chi-square test (or Fisher’s exact test if the expected frequency in any cell was less than 5 and exceeded 20% of the total). The statistical significance was set at one-sided 2.5% and two-sided 5%.

To statistically verify the differences in changes from data measured three times on the same individuals, the Linear Mixed Model (LMM) technique was employed. The LMM is a statistical model used to model relationships between variables, considering the correlation between multiple observations. This model is particularly useful when dealing with correlated or grouped data, such as repeated measurements or hierarchical structures [36].

## 3. Results

### 3.1. Results at Each Time Point for the Overall Evaluation Variables

Through random assignment, considering gender and age, participants were divided into an experimental group of 18 participants (3 males and 15 females, mean age: 64.22 ± 9.67) and a control group of 18 participants (4 males and 14 females, mean age: 65.72 ± 7.82). At the initial screening point, no statistically significant differences were observed between the two groups. Additionally, the distribution of the primary pain complaint area was found to exhibit a similar pattern in both groups.

When analyzing the differences between the two groups at each time point in terms of PPT scores, K-WOMAC scores, and VAS scores, it was observed that the VAS scores significantly decreased in the experimental group as compared to the control group at both 4 and 6 weeks. Although the PPT and K-WOMAC scores showed more improvement in the experimental group, there were no statistically significant differences between the two groups at each time point. These findings suggest that the experimental group experienced a more notable reduction in perceived pain as measured by the VAS; the improvements in pain pressure sensitivity and functional scores (K-WOMAC) were not significantly different from those in the control group shown in Table 4.

### 3.2. Results of Temporal Changes

The analysis of the mean changes in the variables at each time point revealed that, in the case of PPT, no statistically significant differences were observed at any time point. For K-WOMAC, significant differences were observed between baseline (week 0) and week 4, as well as between baseline (week 0) and week 6, but no significant difference was observed between week 4 and week 6. In the case of VAS, there was a significant difference in the mean change between baseline (week 0) and week 4, as well as between baseline (week 0) and week 6 (Table 5).

### 3.3. Results of VAS Using LMM

**Figure 7:**
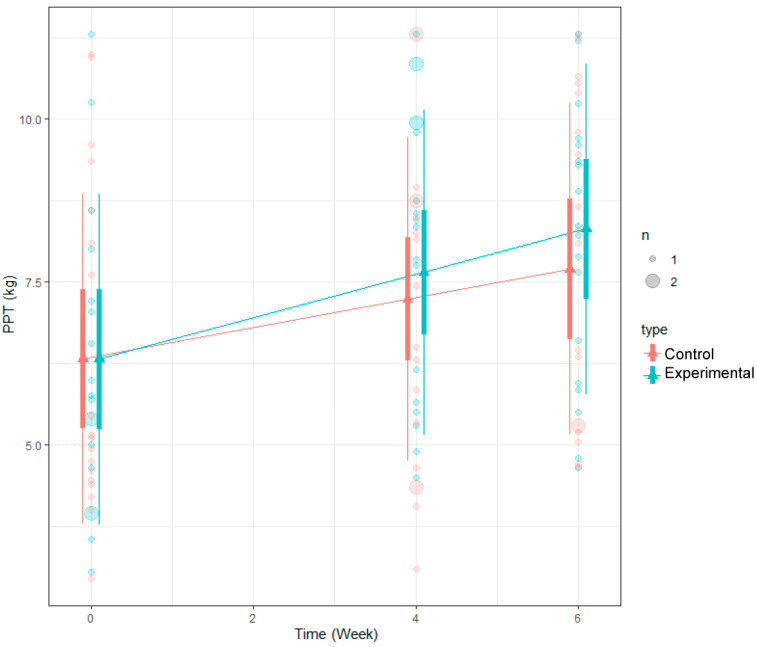
LMM analysis results of PPT.

For the VAS scores, it was found that the scores increased statistically significantly at each of the three time points. Additionally, a significant difference in the averages between the two groups (the experimental group and the control group) was confirmed, with a *p*-value of less than 0.05. This indicates that there was a meaningful change in perceived pain levels over time, and the difference in pain reduction between the two groups was statistically significant (Table 6; Figure 7).

### 3.4. Results of K-WOMAC Using LMM

Regarding the K-WOMAC scores, it was observed that these scores decreased statistically significantly at each of the three time points. However, when comparing the extent of the decrease between the two groups (the experimental group and the control group), although the decrease was not statistically significant, the experimental group showed a greater reduction in K-WOMAC scores (*p*-value: 0.691) (Table 7).

### 3.5. Results of PPT Using LMM

The analysis of changes in PPT using LMM revealed a statistically significant increase in PPT scores at each of the three time points (baseline, 4th week, and 6th week). However, when comparing the magnitude of the increase between the two groups (the experimental group and the control group), although the experimental group exhibited a greater increase in PPT scores compared to the control group, this difference was not statistically significant (*p*-value: 0.6223). These findings suggest that both the experimental and control groups exhibited enhancements in pain sensitivity, as indicated by the increase in PPT scores. However, the discrepancy in the extent of improvement between the two groups did not achieve statistical significance (Table 8).

### 3.6. Anatomical Changes

A comparative analysis of X-ray images captured at the 6-week mark against baseline (week 0) images exhibited no discernible anatomical changes, encompassing spinal tilt and Cobb angle measurements, within both the experimental and control groups.

### 3.7. Analysis Results for Safety Evaluation

Among the 36 participants in this study, 1 reported experiencing abdominal pain, while another reported coughing. The participant with abdominal pain was diagnosed with a gallbladder cyst, while the participant who reported coughing was diagnosed with COVID-19. Both incidents were deemed unrelated to the medical devices used in the clinical trial. Following appropriate medical treatment, both participants were able to continue and complete our study.

## 4. Discussion

Spinal pain is a predominant cause of disability, exhibiting a prevalence of over 60% across all age groups in adults, making it the most common disorder. Furthermore, spinal pain is often accompanied by daily life limitations and discomfort, impacting mental well-being [2,4]. Therefore, appropriate treatment is necessary to enhance the overall quality of life [2,37]. Approaches for alleviating spinal pain encompass both surgical and conservative interventions. Conservative treatments are often preferred for patients with less severe pain or those concerned about the potential side effects of surgery [38]. In various countries across Asia and Eastern Europe, there is widespread utilization of heat and spinal vibration massage devices designed for convenient home use. These devices represent a prevalent form of conservative treatment, specifically heat and massage therapy [14,15].

In previous studies investigating the mechanisms of pain relief through heat and massage, it was demonstrated that heat stimulates thermoreceptors present in both the skin and deep tissues, leading to the closure of the gate in the pain control system, according to the gate control theory [39]. This, in turn, induces a reduction in pain by modulating the pain control system. Massage activates the pain modulation system within the nervous system, fostering recovery through muscle stretching and augmenting the release of pain-reducing mediators [40]. Moreover, it has been shown to increase serotonin and dopamine levels, diminish cortisol, and impart a normalizing effect on motor function. Additionally, it was explained that heat and massage applications increase parasympathetic nervous activity and also lead to an increase in sympathetic nervous activity, so an overall upregulation of autonomic activity is indicated [18].

This study sought to evaluate the clinical significance of pain alleviation, enhancement in daily functioning, and anatomical alterations ensuing from the implementation of heat therapy combined with spinal twisting massage among individuals with chronic non-specific spinal pain, building upon previous research findings in this domain. Additionally, a comparison was made with a control group receiving simple heat and vibration massage to validate the superiority of the experimental group. The spinal twist massage applied to the experimental group involves four massage rollers positioned in the same plane (horizontal) that move three-dimensionally (diagonally) to provide massage. By twisting adjacent vertebrae in opposite directions, it massages to enhance spinal flexibility. The control group received simple heat and vibration massage, providing massage through the horizontal movement of the massage rollers utilizing body pressure. The main results of this study revealed a statistically significant decrease in pain severity when measured by VAS at 4 weeks and 6 weeks compared to the baseline (0 weeks) (*p* < 0.05). The experimental group also showed a statistically significant degree of pain reduction compared to the control group. These findings were obtained by comparing the two groups (*p* < 0.05). In the assessment of daily living function using K-WOMAC, significant decreases in K-WOMAC scores were observed at 4 weeks and 6 weeks compared to the baseline (0 weeks) (*p* < 0.05). Although the comparison between the two groups did not reach statistical significance, the experimental group exhibited a larger decrease in K-WOMAC scores. This suggests that heat and spinal twisting massage not only have an effect on pain relief but also contribute to improvements in physical activity. The analysis of changes in PPT scores at each time point revealed a statistically significant increase (*p* < 0.05). Also, the experimental group showed a greater increase compared to the control group, although the difference was not statistically significant. Similar to previous studies that demonstrated the efficacy of thermal massage devices in relieving pain and improving mood and stress [17,18], the present study also confirmed the effectiveness of applying heat and spinal twist massage for alleviating pain and enhancing functional outcomes in patients with chronic non-specific spinal pain [17,18,19,21,22]. Remarkably, the outcomes demonstrated a greater effect of heat and spinal twist massage in comparison to earlier studies that employed traditional heat or basic vibration massage. Furthermore, this study did not exhibit the side effects, such as localized soreness and discomfort, that were noted in So et al.’s investigation of commonly used heat and massage devices in Asia, the Middle East, and Europe [23].

The study results also revealed that short-term treatment with heat and spinal twisting massage may not induce anatomical structural changes. However, it appears to be more effective in relieving pain for patients with chronic non-specific spinal pain compared to simple vibration and heat massage. Combining spinal twisting massage with simple vibration massage could indeed offer potential benefits for individuals dealing with chronic non-specific spinal pain. Spinal twisting can help improve flexibility and mobility in the spine, while vibration massage can aid in relaxation and alleviate muscle tension. By integrating these techniques, therapists may enhance their therapeutic effects and offer a more comprehensive approach to managing spinal pain. However, this study has several limitations. Our study did not investigate long-term consequences or potential variations in treatment response among different patient subgroups, even though it showed considerable pain reduction and functional improvement with heat and spine twisting massage for chronic non-specific spinal pain. Furthermore, this study did not compare the effectiveness of spinal twisting massage and heat therapy to other conservative therapies or interventions that went beyond simple vibration massage.

## 5. Conclusions

This study aimed to investigate the clinical effects of heat and spinal twist massage on patients with chronic non-specific spinal pain. Due to limitations in subject recruitment, study duration, and costs, the target age group was restricted, and anatomical changes could not be observed. Furthermore, a shortcoming of the focus on clinical improvement was the neglect of physiological and biochemical processes. However, the study results demonstrate the potential of spinal twist massage as an innovative therapeutic tool for pain relief and enhancement of daily functioning in patients with chronic non-specific spinal pain. Future investigations may incorporate quantitative analysis and simulation methodologies to delve deeper into the optimal direction and intensity of stimuli necessary to maximize the therapeutic effects of spinal twist massage. Moreover, long-term clinical trials, encompassing diverse participant age groups, expanded sample sizes, and variations in treatment frequency and incorporating research on physiological and biochemical mechanisms, are anticipated to yield further enhancements in treatment outcomes.

## Figures and Tables

**Table 1:** Characteristics of participants.

Category	Total
Age	
Mean (SD)	64.97 (8.7)
Min–Max	40–78
Median (IQR)	67 (9)
Gender	
Male	7 (19.44)
Female	29 (80.56)

**Table 2:** Inclusion and exclusion criteria.

Inclusion Criteria	Exclusion Criteria
➢ Participants diagnosed with chronic non-specific spinal pain.	➢ History of spinal procedures or surgery within the last 3 months.
➢ Age between 20 and 80 years.	➢ Chronic inflammatory conditions such as ankylosing spondylitis.
➢ VAS score of at least 40 mm (maximum 100 mm).	➢ Potential interference with treatment efficacy due to other diseases.
➢ K-WOMAC score of 30 or higher.	➢ Patients with limb dissymmetry were not excluded from this research.

**Table 3:** Characteristics of cont. group vs. exp. group.

Category	Total	Cont.	Exp.	*p*-Value
Age				0.6221
Mean (SD)	64.97 (8.7)	65.72 (7.82)	64.22 (9.67)	
Min–Max	40–78	45–78	40–78	
Median (IQR)	67 (9)	67 (8)	67.5 (9)	
Gender				0.6737
Male	7 (19.44)	4 (22.22)	3 (16.67)	
Female	29 (80.56)	14 (77.78)	15 (83.33)	

**Table 4:** Overall variable characteristics.

Category	Cont.	Exp.	*p*-Value
At baseline (week 0)			
PPT mean (SD) (kg)	6.4 (2.57)	6.19 (2.25)	0.7921
K-WOMAC mean (SD) (score)	50.22 (11.89)	55.06 (10.71)	0.2089
VAS mean (SD) (mm)	60.06 (8.88)	64.44 (12.95)	0.2439
After treatment (week 4)			
PPT mean (SD) (kg)	6.99 (2.43)	8.02 (2.22)	0.1940
K-WOMAC mean (SD) (score)	41.67 (17.33)	32.61 (12.73)	0.0829
VAS mean (SD) (mm)	47.56 (15.27)	35.22 (10.52)	0.0079 *
Follow-Up (week 6)			
PPT mean (SD) (kg)	7.86 (2.48)	8.06 (2.1)	0.7954
K-WOMAC mean (SD) (score)	35.72 (18.12)	27.5 (13.24)	0.1293
VAS mean (SD) (mm)	46.94 (17.88)	31.56 (11.89)	0.0045 *

Note: * *p* < 0.05 is significant at α = 0.05. Values are presented as mean (standard deviation); Cont. = control; Exp. = experimental; PPT = Pressure Pain Threshold; K-WOMAC = Korean version of the Western Ontario and McMaster Universities Osteoarthritis Index; VAS = Visual Analog Scale.

**Table 5:** Changes in mean value from baseline (week 0) to weeks 4 to 6.

	Week 4 to Baseline			Week 6 to Week 4			Week 6 to Baseline		
	Cont.	Exp.	*p*-Value	Cont.	Exp.	*p*-Value	Cont.	Exp.	*p*-Value
PPT (kg)	0.59 (2.07)	1.84 (2.56)	0.118	0.86 (1.58)	0.04 (1)	0.069	1.46 (2.13)	1.87 (2.23)	0.572
K-WOMAC (score)	−8.56 (14.77)	−22.44 (9.21)	0.001 *	−5.94 (14.11)	−5.11 (8.36)	0.830	−14.5 (11.33)	−27.56 (10.25)	0.001 **
VAS (mm)	−12.5 (16.82)	−29.22 (13.02)	0.002 *	−0.61 (11.39)	−3.67 (5.43)	0.314	−13.11 (16.43)	−32.89 (14.01)	0.001 **

Note: * *p* < 0.05 and ** *p* < 0.001 are significant at α = 0.05. Values are presented as mean (standard deviation); Cont. = control; Exp. experimental; PPT = Pressure Pain Threshold; K-WOMAC = Korean version of the Western Ontario and McMaster Universities Osteoarthritis Index; VAS = Visual Analog Scale.

**Table 6:** Changes in VAS from baseline (week 0) to weeks 4 to 6.

VAS (mm)	Estimated Mean	T Value	*p*-Value
Cont.	51.52 (2.53)	20.35	<0.0001 **
Exp.	43.74 (2.53)	17.28	<0.0001 **
Cont. vs. Exp.	7.78 (3.58)	2.17	0.0369 *

Note: * *p* < 0.05 and ** *p* < 0.001 are significant at α = 0.05. Values are presented as mean (standard deviation); VAS = Visual Analog Scale; Cont. = control; Exp = experimental.

**Table 7:** Changes in K-WOMAC from baseline (week 0) to weeks 4 to 6.

K-WOMAC (Score)	Estimated Mean	T Value	*p*-Value
Cont.	42.97 (2.99)	14.38	<0.0001 **
Exp.	41.28 (2.99)	13.81	<0.0001 **
Cont. vs. Exp.	1.69 (4.23)	0.4	0.691

Note: ** *p* < 0.001 are significant at α = 0.05. Values are presented as mean (standard deviation); K-WOMAC = Korean version of the Western Ontario and McMaster Universities Osteoarthritis Index; Cont. = control; Exp. = experimental.

**Table 8:** Changes in PPT from baseline (week 0) to weeks 4 to 6.

PPT (kg)	Estimated Mean (SE)	T Value	*p*-Value
Cont.	7.08 (0.48)	14.7	<0.0001 **
Exp.	7.42 (0.48)	15.4	<0.0001 **
Cont. vs. Exp.	−0.34 (0.68)	−0.5	0.6233

Note: ** *p* < 0.001 are significant at α = 0.05. Values are presented as mean (standard deviation); PPT = Pressure Pain Threshold; Cont. = control; Exp = experimental.

## Data Availability

Data are contained within the article.
